# Parallel progressive multiple sequence alignment on reconfigurable meshes

**DOI:** 10.1186/1471-2164-12-S5-S4

**Published:** 2011-12-23

**Authors:** Ken D Nguyen, Yi Pan, Ge Nong

**Affiliations:** 1 Department of Information Technology, Clayton State University, Morrow, GA 30260, USA; 2 Department of Computer Science, Georgia State University, Atlanta, GA 30303, USA; 3 Department of Computer Science, Sun Yat-sen University, P.R.C

## Abstract

**Background:**

One of the most fundamental and challenging tasks in bio-informatics is to identify related sequences and their hidden biological significance. The most popular and proven best practice method to accomplish this task is aligning multiple sequences together. However, multiple sequence alignment is a computing extensive task. In addition, the advancement in DNA/RNA and Protein sequencing techniques has created a vast amount of sequences to be analyzed that exceeding the capability of traditional computing models. Therefore, an effective parallel multiple sequence alignment model capable of resolving these issues is in a great demand.

**Results:**

We design *O*(1) run-time solutions for both local and global dynamic programming pair-wise alignment algorithms on reconfigurable mesh computing model. To align *m *sequences with max length *n*, we combining the parallel pair-wise dynamic programming solutions with newly designed parallel components. We successfully reduce the progressive multiple sequence alignment algorithm's run-time complexity from *O*(*m *× *n*^4^) to *O*(*m*) using *O*(*m *× *n*^3^) processing units for scoring schemes that use three distinct values for match/mismatch/gap-extension. The general solution to multiple sequence alignment algorithm takes *O*(*m *× *n*^4^) processing units and completes in *O*(*m*) time.

**Conclusions:**

To our knowledge, this is the first time the progressive multiple sequence alignment algorithm is completely parallelized with *O*(*m*) run-time. We also provide a new parallel algorithm for the Longest Common Subsequence (LCS) with *O*(1) run-time using *O*(*n*^3^) processing units. This is a big improvement over the current best constant-time algorithm that uses *O*(*n*^4^) processing units.

## Background

The advancement of DNA/RNA and protein sequencing and sequence identification has created numerous databases of sequences. One of the most fundamental and challenging tasks in bio-informatics is to identify related sequences and their hidden biological significance. Aligning multiple sequences together provides researchers with one of the best solutions to this task. In general, multiple sequence alignment can be defined as:

Definition 1

*Given: m sequences, (s*_1_*, s*_2_,..., *s*_*m*_), *over an alphabet *∑, *where each sequence contains up to n symbols from *∑*; a scoring function h*: ∑×∑×⋯×∑→ℜ; *and a gap cost function. Multiple sequence alignment is a technique to transform (s*_1_, *s*_2_, ..., *s*_*m*_) *to *$\left(s_{1}^{\prime },s_{2}^{\prime },\cdots \;,s_{m}^{\prime }\right)$, *where *$s_{i}^{\prime }$*is s*_*i *_∪ '-' *[gap insertions], that optimizes the matching scores between the residues across all sequence columns *[1]. However, multiple sequence alignment is an NP-Complete problem [2]; therefore, it is often solved by heuristic techniques. Progressive multiple sequence alignment is one of the most popular multiple sequence alignment techniques, in which the pair-wise symbol matching scores can be derived from any scoring scheme or obtained from a substitution scoring matrix such as PAM [3] or BLOSUM [4]. There are many implementations of progressive multiple sequence alignment as seen in [5-8]. In general, progressive multiple sequence alignment algorithm follows three steps:

(i) Perform all pair-wise alignments of the input sequences.

(ii) Compute a dendrogram indicating the order in which the sequences to be aligned.

(iii) Pair-wise align two sequences (or two pre-aligned groups of sequences) following the dendrogram starting from the leaves to the root of the dendrogram.

**Figure 1:**
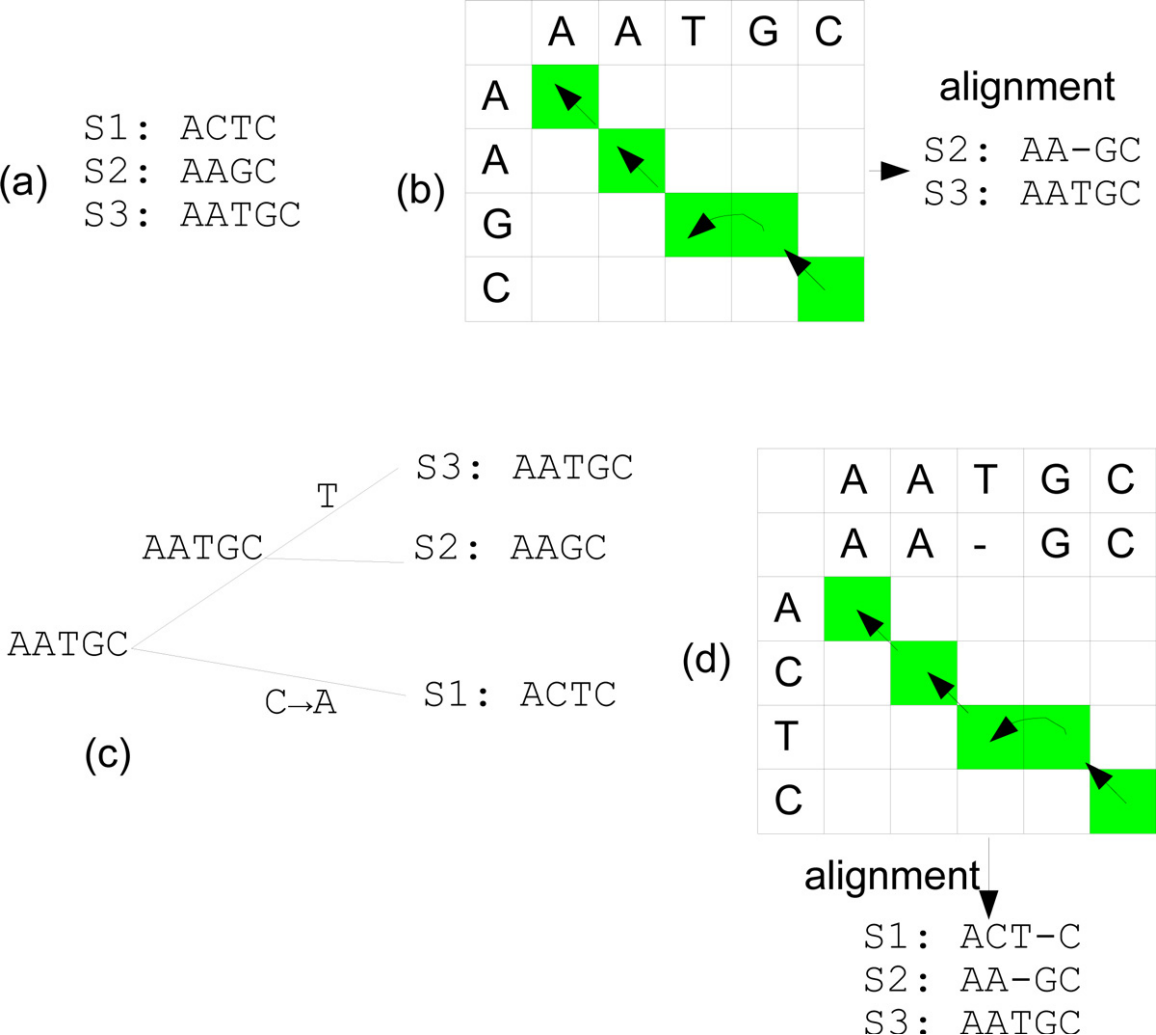
**A progressive multiple sequence alignment**. An example of progressive Multiple Sequence Alignment. (a) represents three input sequences (S1, S2, S3); (b) shows the pair-wise dynamic programming alignment of two sequences; (c) shows the order of the sequences to be aligned, where the leaves on right hand-side are the input sequences, the internal nodes represent the theoretical ancestors from which the sequences are derived, and the characters on the tree branches represent the missing/mutated residues; and (d) shows the pair-wise dynamic programming of two pre-aligned groups of sequences.

Figure 1 shows an example of these steps, where (a) represents the input sequences, (b) represents an alignment of step (i), (c) shows the dendrogram obtained from step (ii), and (d) shows a pair-wise group-alignment in step (iii).

Step (i) can be optimally solve by Dynamic Programming (DP) algorithm. There are two versions of DP: the Smith-Waterman's [9] is used to find the optimally aligned segment between two sequences (local DP), and the Needleman-Wunsch's [10] is used to find the global optimal overall sequence pair-wise alignment (global DP). The two algorithms are very similar and will be described in more details in the next section. The dynamic programming algorithms take *O*(*n*^2^) time to complete, including the back-tracking steps. Thus, with $\frac{m\left(m-1\right)}{2}$ unique pairs of the input sequences, the run-time complexity of step (i) is *O*(*m*^2 ^*n*^2^) or *O*(*n*^4^) if *n *and *m *are asymptotically equivalent.

To generate a dendrogram from the distances between the sequences (or the scores generated from step (i)), either UPGMA [11] or Neighbor Joining (NJ) [12] hierarchical clustering is used. These algorithms yield *O*(*m*^3^) run-time complexity.

In the worst case, step (iii) performs (*m *- 1) pair-wise alignments in-order following the dendrogram hierarchy. Similar to step (i), dynamic programming for pair-wise alignment is used, however, each of these pair-wise group alignment yields an order of *O*(*n*^4^) via dynamic programming (*O*(*n*^2^)) and sum-of-pair scoring function [13](*O*(*n*^2^)). This scoring function is required to evaluate every all possible residue matchings of the sequences. As a result, the run-time complexity of step (iii) is *O*(*m *× n^4^) ≈ *O*(*n*^5^), which is the overall run-time complexity of progressive multiple sequence alignment algorithm.

### Optimal pair-wise sequence alignment by dynamic programming

Given two sequences *x *and *y *each contains up to *n *residue symbols. The optimal alignment of these sequences can be found by calculating an (*n *+ 1) × (*n *+ 1) dynamic programming (DP) matrix containing all possible pair-wise matching scores of residue symbols in the sequences. Initially, the first row and column of the matrix cells are set to 0, i.e.

$$\begin{array}{c} c_{0,j}=0,\\ c_{i,0}=0. \end{array}$$

The recursive formula to compute the DP matrix for the Longest Common Subsequence (LCS) as seen in [14] is:

$$c_{i,j}=\left\{\begin{array}{cc} c_{i-1,j-1}+1 & \text{if}\;x_{i}=y_{j}\\ max\left\{\left(c_{i-1,j}\right),\left(c_{i,j-1}\right)\right\} & \text{if}\;x_{i}\neq y_{j} \end{array}\right)$$

Similarly, the Needleman-Wunsch's algorithm [10] uses the following formula to complete the DP matrix:

$$c_{i,j}=max\left\{\begin{array}{cc} c_{i-1,j-1}+s\left(x_{i},y_{j}\right) & \text{symbol matching}\\ c_{i-1,j}+g & \text{gap insertion}\\ c_{i,j-1}+g & \text{gap insertion} \end{array}\right)$$

where s(*x*_*i*_, *y*_*j*_) is the pair-wise symbol matching score of the two symbols *x*_*i *_and *y*_*j *_from sequences *x *and *y*, respectively; and *g *is the gap cost for extending a sequence by inserting a gap, i.e. gap insertion/deletion (indel).

Smith and Waterman [9] modified the above formula as:

$$c_{i,j}=max\left\{\begin{array}{cc} 0 & \\ c_{i-1,j-1}+s\left(x_{i},y_{j}\right) & \text{symbol matching}\\ c_{i-1,j}+g & \text{gap insertion}\\ c_{i,j-1}+g & \text{gap insertion} \end{array}\right)$$

The alignment can be obtained from the DP matrix by starting from cell *c*_*n*, *n*_, (or the cell containing the max value in the matrix as in the Smith-Waterman's algorithm), and tracking back to the top of the matrix, i.e. cell c_0,0_, by following neighboring cells with the largest value.

### Existing parallel implementations

Progressive multiple sequence alignment algorithms are widely parallelized, mostly because they perform $\frac{m\left(m-1\right)}{2}$ independent pair-wise alignments as in step (i). These individual pair-wise alignments can be designated to different processing units for computation as in [15-24]. These implementations are across many computing architectures and platforms. For example, [17] implemented a DP algorithm on Field-Programmable Gate Array (FPGA). Similarly, Oliver et al. [23,24] distributed the pair-wise alignment of the first step in the progressive alignment, where all pair-wise alignments are computed, on FPGA. Liu et al. [18] computed DP via Graphic Processing Units (GPUs) using CUDA platform, [22] used CRCW PRAM neural-networks, [15] used Clusters, [16] used 2D r-mesh, [20] used Network mesh, or [21] used 2D Pr-mesh computing model.

The two most notable parallel versions of dynamic programming algorithm are proposed by Huang [25] and Huang et al. and Aluru [15,26]. Huang's algorithm exploits the independency between the cells on the anti-diagonals of the DP matrix, where they can be calculated simultaneously. There are 2*n *+ 1 anti-diagonals on a matrix of size (*n *+ 1 × *n*+1). Thus, this parallel DP algorithm takes *O*(*n*) processing units and completes in *O*(*n*) time.

Independently, Huang et al. [15] and Aluru et al. [26] propose similar algorithms to partition the DP matrix column-wise and assign each partition to a processor. Next, all processors are synchronized to calculate their partitions one row at a time. For this algorithm to perform properly, each processor must hold a copy of the sequence that mapped to the rows of the matrix. Since these calculations are performed row-wise, the values from cells *c*_*i*-1, *j*-1 _and *c*_*i*-1, *j *_are available before the calculation of cell *c*_*i,j*_. The value of *c*_*i*, *j*-1 _can be obtained by performing prefix-sum across all cells in row *i*^*th*^. Thus, with *n *processors, the computation time of each row is dominated by the prefix-sum calculations, which is *O*(*logn*) time on PRAM models. Therefore, the DP matrix can be completed in *O*(*nlogn*) time using *O*(*n*) processors. Recently, Sarkar, et al. [19] implement both of these parallel DP algorithms [25,26] on a Network-on-Chip computing platform [27].

In addition, the construction of a dendrogram can be parallelized as in [18] using *n *Graphics Processing Units (GPUs) and completing in *O*(*n*^3^) time.

Furthermore, there are attempts to parallelize the progressive alignment step [step (iii)] as in [28] and [29]. In [28], the independent pre-aligned pairs along the dendrogram are aligned simultaneously. This technique gains some speed-up, however, the time complexity of the algorithm remains unchanged since all the pair-wise alignments eventually must be merged together. Another attempt is seen in [29], where Huang's algorithm [25] is used. When an anti-diagonal of a DP alignment matrix in lower tree level in step (iii) is completed, it is distributed immediately to other processors for computing the pair-wise alignment of a higher tree level. This technique can lead to an incorrect result since the actual pair-wise alignment of the lower branch is still uncertain.

Overall, the major speedups achieved from these implementations come from two parallel tasks: performing $\frac{m\left(m-1\right)}{2}$ initial pair-wise alignments in step (i) simultaneously and calculating the dynamic programming matrix anti-diagonally (or in blocks). These tasks potentially can lower the run-time complexity of step (i) from *O*(*m*^2^*n*^2^) to *O*(*n*) and step (iii) from *O*(*mn*^4^) to *O*(*m*^3^*n*) ≈ *O*(*n*^4^), [or *O*(*m*^4^) if *n *<*m*]. The overall run-time complexity of the original progressive multiple sequence alignment algorithm is still dominated by step (iii) with an order of *O*(*m*^3^*n*) regardless of how many processing units are used. The bottle-neck is the pair-wise group alignments must be done in order dictated by the dendrogram (*O*(*m*)), and each alignment requires all the column pair-wise scores be calculated (*O*(*m*^2^)). To address these issues, we design our parallel progressive multiple sequence alignment on a reconfigurable mesh (r-mesh) computing model similar to the ones used in [16,23,24]. Following is the detailed description of the r-mesh model.

### Reconfigurable-mesh computing models - (r-mesh)

**Figure 2:**
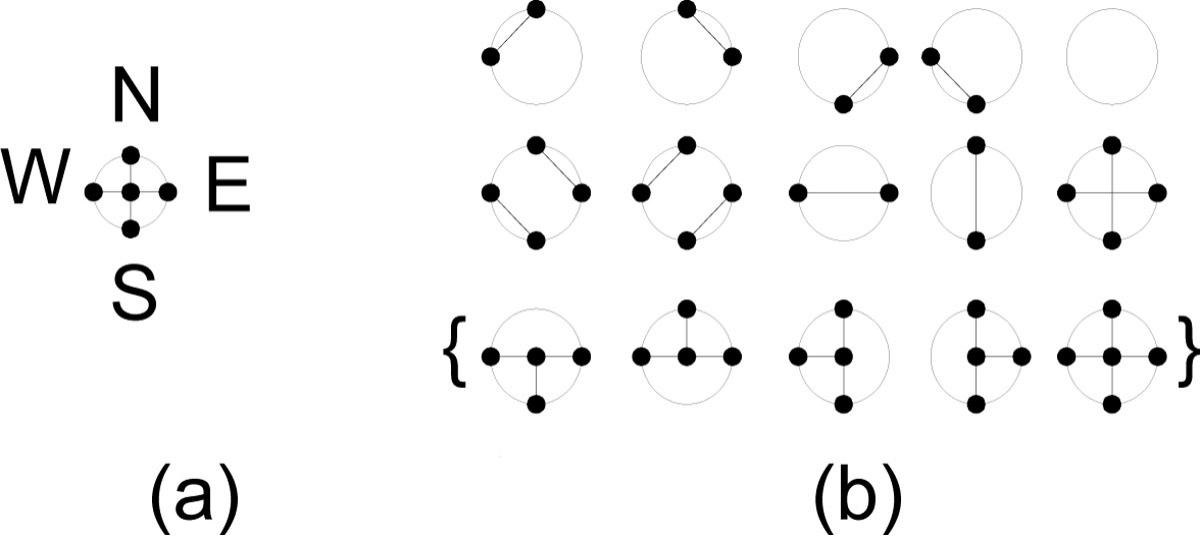
**Port configurations on reconfigurable computing model**. Allowable configurations on 4 port processing units; (a) shows the ports directions; (b) shows the 15 possible port connections, where the last five port configurations in curly braces are not allowed in Linear r-mesh (Lr-mesh) models.

A Reconfigurable mesh (r-mesh) computing, first proposed by Miller et al [30], is a two-dimensional grid of processing units (PUs), in which each processing unit contains 4 ports: North, South, East, and West (N, S, E, W). These ports can be fused or defused in any order to connect one node of the grid to its neighboring nodes. These configurations are shown in Figure 2. Each processing unit has its own local memory, can perform simple arithmetic operations, and can configure its ports in O(1) time.

There are many reconfigurable computing models such as Linear r-mesh (Lr-mesh), Processor Array with Reconfigurable Bus System (PARBS), Pipedlined r-mesh (Pr-mesh), Field-programmable Gate Array (FPGA), etc. These models are different in many ways from construction to operation run-time complexities. For example, the Pr-mesh model does not function properly with configurations containing cycles, while many other models do. However, there are many algorithms to simulate the operations of one reconfigurable model onto another in constant time as seen in [31-36].

In the scope of this study, we will use a simple electrical r-mesh system, where each processing unit, or processing element (PU or PE), contains four ports and can perform basic routing and arithmetic operations. Most reconfiguration computing models utilize the representation of the data to parallelize their operations; and there are various proposed formats [37]. Commonly, data in one format can be converted to another in *O*(1) time [37]. The unary representation format is used this study, which is denoted as 1UN, and is defined as:

Definition 2

*Given an integer x *∈ [0, *n *- 1]*, the unary 1UN presentation of x in n-bit is: x *= (*b*_0_, *b*_1_, ..., *b*_*n*__-1_)*, where b*_*i *_= 1 *for all i *≤ *x and b*_*i *_= 0 *for all i > x *[37].

For example, a number 3 is represented as 11110000 in 8-bit 1UN representation.

In addition to the 1UN unary format, we will be utilizing the following theorem for some of the operations:

**Theorem 1**:

*The prefix-sum of n value in range *[0, *n*^*c*^] *can be found in O*(*c*) *time on an n *× *n r-mesh *[37].

In terms of multiple sequence alignment, the number of bits used in the 1UN notation is correlated to the maximum length of the input sequences. In the next Section, we will describe the designs of r-meshcomponents to use in dynamic programming algorithms.

## Parallel pair-wise dynamic programming algorithms

This section begins with the description of several configurations of r-mesh needed to compute various operations in pair-wise dynamic programming algorithm. Following the r-mesh constructions is a new constant-time parallel dynamic programming algorithm for Needleman-Wunsch's, Smith-Waterman, and the Longest Common Subsequence (LCS) algorithms.

### R-mesh max switches

**Figure 3:**
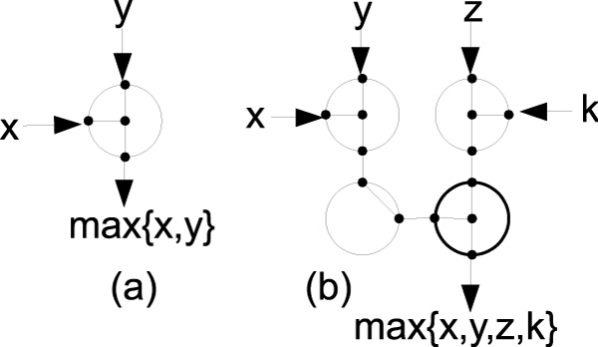
**1-bit max switches**. Two 1-bit max switches. (a)- fusing {NSEW} to find the max of two inputs from North and West ports; (b)- construction of a 1-bit 4-input max switch.

**Figure 4:**
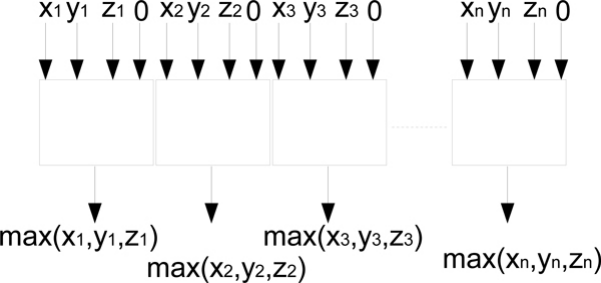
**An n-bit 3-input max switch**. An n-bit 3-input max switch, where the rectangle represents a 1-bit 4-input max switch from Figure 3.

One of the operations in the dynamic programming algorithm requires the capability to select the largest value from a set of input numbers. Following is the design of an r-mesh switch that can select the maximum value from an input triplet in the same broadcasting step. For 1-bit data, the switch can be built as in Figure 3(a) using one processing unit, (introduced by Bertossi [14]). The unit configures its ports as {NSEW}, where the North and West are input ports and the others are output ports. When a signal (or 1) comes through the switch, the max bit will propagate through the output ports. Similarly, a switch for finding a maximum value of four input bits can be built using 4 processing units with configurations: {NSW,E}, {NSE,W}, {NE,S,W}, and {NSW,E} as in Figure 3(b). To simulate a 3-input max switch on positive numbers, one of the input ports loads in a zero value. These switches can be combined together to handle the max of three n-bit values as in Figure 4. This n-bit max switch takes 4 × *n*, (i.e. *O*(*n*)) processing units and can handle 3 to 4 n-bit input numbers. All of these max switches allow data to flow directly through them in exactly one broadcasting step. They will be used in the design of our algorithm, described latter.

### R-mesh adder/subtractor

**Figure 5:**
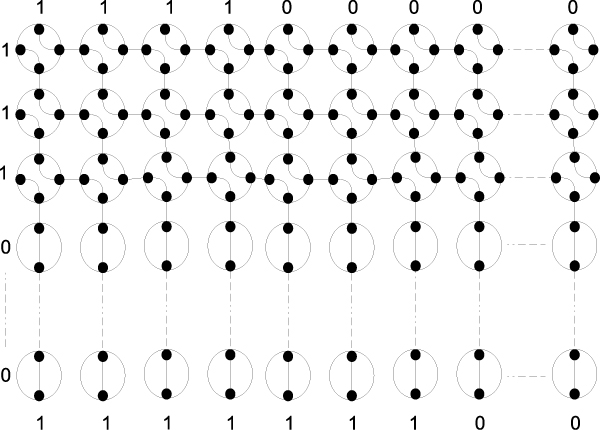
**An n-bit adder/subtractor**. An n-bit adder/subtractor that can perform addition or subtraction between two 1UN numbers during a broadcasting time. For additions the inputs are on the North and West borders and the output is on the South border. For subtractions, the inputs are on the West and South borders and the output is on the North border. The number on the West bound is 1-bit left-shifted. The dotted lines represent the omitted processing units that are the same as the ones in the last rows. This figure shows the addition of 3 and 3. Note: the leading 1 bit of input number on the West-bound (left) has been shifted off. The right border is fed with zero (or no signal) during the subtract operation.

Similarly, to get a constant time dynamic programming algorithm we have to be able to perform a series of additions and subtractions in one broadcasting step. Exploiting the properties of 1UN representation, we are presenting an adder/subtractor that can perform an addition or a subtraction of two n-bit numbers in 1UN representation in one broadcasting time. The adder/subtractor is a *k *× *n *r-mesh, where *k *is the smaller magnitude of the two numbers. The r-mesh adder/subtractor is shown in Figure 5. To perform addition, one addend is fed into the North-bound of the r-mesh, and another addend is left-shifted one bit and fed into the West-bound. The left-bit shifting operation removes the bit that represents a zero, which in turn reduces one row of the r-mesh. Similarly, there is no need to have extra rows in the r-mesh to perform additions on the right trailing zeros of the second addend. Therefore, the number of rows in the r-mesh adder/subtractor can be reduced to *k*, where *k *+ 1 is the number of 1-bits in the second addend. Each processing unit in the adder/subtractor fuses {NE, SW} if the West input is 1, otherwise, it will fuse {NS, E, W}. The first configuration allows the number to be incremented if there is a 1-bit coming from the West, and the second configuration maps the result directly to the output ports. Figure 5 shows the addition of 3 and 3 represented in n-bit 1UN. In this case, the r-mesh needs only 3 rows to compute the result. Similarly, for subtractions, the minuend is fed into the South bound (bottom) of the r-mesh, the subtrahend is 1-bit left-shifted and fed into the r-mesh from the West bound (left), the East bound (right) is fed with zeros, or no signals. The output is obtained from the North border (top).

This adder/subtractor can only handle numbers in 1UN representation, i.e. positive values. Thus, any operation that yields a negative result will be represented as a pattern of all zeros. When this adder/subtractor is used in a DP algorithm, one of the two inputs is already known. For example, to calculate the value at cell *c*_*i*, *j*_, three binary arithmetic operations must be performed: *c*_*i*-1, *j*-1 _+ *s*(*x*_*i*_, *y*_*j*_), *c*_*i*-1, *j *_+ *g*, and *c*_*i*, *j*-1 _+ *g*, where both the gap *g *and the symbol matching score *s*(*x_i_*, *y_j_*) between any two residue symbols are predefined. Thus, we can store these predefined values to the West ports of the adder/subtractor units and have them configured accordingly before the actual operations.

For biological sequence alignments, symbol matching scores are commonly obtained from substitution matrices such as PAM [3], BLOSUM [4], or similar matrices, and gap cost is a small constant in the same range of the values in these matrices. These values are one or two digits. Thus, *k *is very likely is a 2-digit constant or smaller. Therefore, the size of the adder/subtractor unit is bounded by *O*(*n*), in this scenario.

### Constant-time dynamic programming on r-mesh

The dynamic programming techniques used in the Longest Common Subsequence (LCS), Smith-Waterman's and Needle-Wunsch's algorithms are very similar. Thus, a DP r-mesh designed to solve one problem can be modified to solve another problem with minimal configuration. We are presenting the solution for the latter cases first, and then show a simple modification of the solution to solve the first case.

#### Smith-Waterman's and Needle-Wunsch's algorithms

Although the number representation can be converted from one format to another in constant time [37], the DP r-mesh run-time grows proportionally with the number of operations being done. These operations could be as many as *O*(*n*^2^). To eliminate this format conversion all the possible symbol matching scores, or scoring matrix, (4 × 4 for RNA/DNA sequences and 20 × 20 for protein sequences) are pre-scaled up to positive values. Thus, an alignment of any pair of residue symbols will yield a positive score; and gap matching (or insert/delete) is the only operation that can reduce the alignment score in preceding cells. Nevertheless, if the value in cell *c*_*i*-1, *j *_(or *c*_*i,j*-1_) is smaller than the magnitude of the gap cost (|*g*|), a gap penalized operation will produce a bit pattern of all zeros (an indication of an underflow or negative value). This value will not appear in cell *c*_*i,j *_since the addition of the positive value in cell *c*_*i*-1, *j*-1 _and the positive symbol matching score *s*(*x*_*i*_, *y*_*i*_) is always greater than or equal to zero.

In general, we do not have to perform this scale-up operation for DNA since DNA/RNA scoring schemes that generally use only two values: a positive integer value for match and the same cost for both mismatch and gap.

Unlike DNA, scoring protein residue alignment is often based on scoring scoring/substitution/mutation matrices such as that in [3,4]. These matrices are log-odd values of the probabilities of residues being mutated (or substituted) into other residues. The difference between the matrices are the way the probabilities being derived. The smaller the probability, the less likely a mutation happens. Thus, the smallest alignment value between any two residues, including the gap is at least zero. To avoid the complication of small positive fractional numbers in calculations, log-odd is applied on these probabilities. The log-odd score or substitution score in [3] is calculated as $s\left(i,j\right)=\frac{1}{\lambda }log\left(\frac{Q_{ij}}{P_{i}P_{j}}\right)$, where *s*(*i*, *j*) is the substitution score between residues *i *and *j*, *λ *is a positive scaling factor, *Q*_*ij *_is the frequency or the percentage of residue *i *correspond to residue *j *in an accurate alignment, and *P*_*i *_and *P*_*j *_are background probabilities which residues *i *and *j *occur. These probabilities and the log-odd function to generate the matrices are publicly available via The National Center for Biotechnology Information's web-site (http://www.ncbi.nlm.nih.gov) along with the substitution matrices themselves. With any given gap cost, the probability of a residue aligned with a gap can be calculated proportionally from a given gap cost and other values from the un-scaled scoring matrices by taking anti-log of the log-odd values or score matrix. Thus, when a positive number *β *is added to the scores in these scoring matrices, it is equivalent to multiply the original probabilities by *a*^*β*^, where *a *is the log-based used in the log-odd function.

A simple mechanism to obtain a scaled-up version of a scoring matrix is: (a) taking the antilog of the scoring matrix and *g*, where *g *is the gap costs, i.e. the equivalent log-odd of a gap matching probability; (b) multiplying these antilog values by *β *factor such that their minimum log-odd value should be greater than or equal to zero; (c) performing log-odd operation on these scaled-up values.

When these scaled-up scoring matrices are used, the Smith-Waterman's algorithm must be modified.

Instead of setting sub-alignment scores to zeros when they become negative, these scores are set to *β *when they fall below the scaled-up factor (*β*).

Using scaled-up scoring matrices will eliminate the need for signed number representation in our following algorithm designs. However, if there is a need to obtain the alignment score based on the original scoring matrices, the score can be calculated as follows: (i) load the original score matrix and gap cost to each cell on an r-mesh as similar to the one described in Section; (ii) configure cells on the diagonal path to use their corresponding matching score from the matrix and other cells representing gap insertions or deletions to use gap cost; (iii) calculate the prefix-sum of all the cells on the path representing the alignment using Theorem 1.

**Figure 6:**
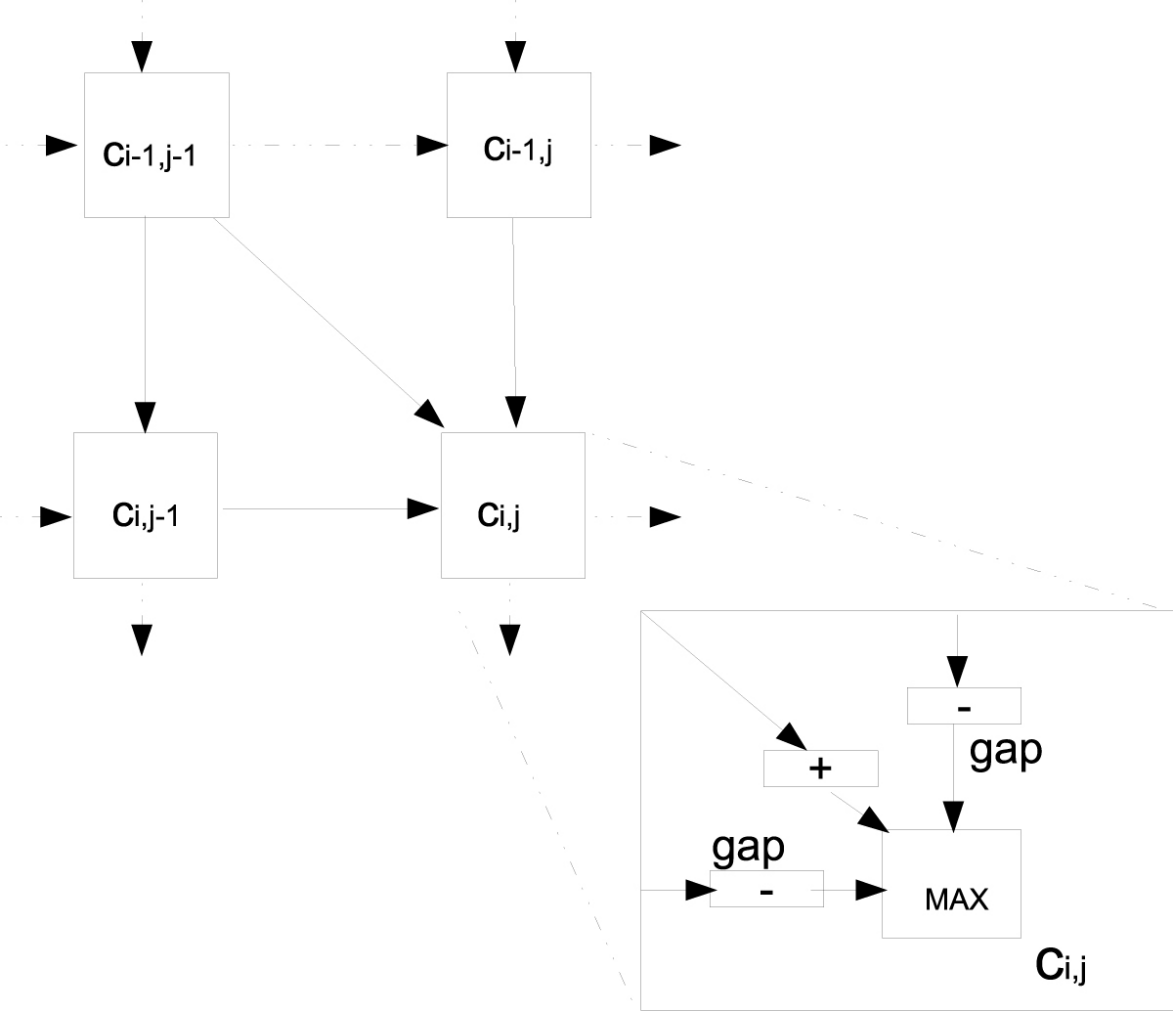
**A dynamic programming r-mesh**. Each cell *c*_*i*, *j *_is a combination of a 3-input max switch and three adder/subtractor units. The "+" and "-" represent the actual functions of the adder/subtractor units in the configuration.

Having the adder/subtrator units and the switches ready, the dynamic programming r-mesh, (DP r-mesh), can be constructed with each cell *c*_*i,j *_in the DP matrix containing 3 adder/subtractor units and a 3-input max switch allowing it to propagate the max value of cells *c*_*i*-1, *j*-1_, *c*_*i*-1, *j *_and *c*_*i*, *j*-1 _to cell c_*i*, *j *_in the same broadcasting step. Figure 6 shows the dynamic programming r-mesh construction. The adder/subtrator units are represented as "+" or "-" corresponding to their functions.

A 1 × n adder/subtractor unit can perform increments and decrements in the range of [-1,0,1]. As a result, a DP r-mesh can be built with 1-bit input components to handle all pair-wise alignments using constant scoring schemes that can be converted to [-1,0,1] range. For instance, the scoring scheme for the longest common subsequence rewards 1 for a match and zero for mismatch and gap extension.

To align two sequences, *c*_*i*, *j *_loads or computes its symbol matching score for the symbol pair at row *i *column *j*, initially. The next step is to configure all the adder/subtractor units based on the loaded values and the gap cost *g*. Finally, a signal is broadcasted from *c*_0,0 _to its neighboring cells *c*_0,1_, *c*_1,0_, and *c*_1,1 _to activate the DP algorithm on the r-mesh. The values coming from cells *c*_*i*-1, *j *_and *c*_*i*, *j*-1 _are subtracted with the gap costs. The value coming from *c*_*i*-1, *j*-1 _is added with the initial symbol matching score in *c*_*i*, *j*_. These values will flow through the DP r-mesh in one broadcasting step, and cell *c*_*n*, *n *_will receive the correct value of the alignment.

In term of time complexity, this dynamic programming r-mesh takes a constant time to initialize the DP r-mesh and one broadcasting time to compute the alignment. Thus, its run-time complexity is *O*(1). Each cell uses 10*n *processing units (4*n *for the 1-bit max switch and 2*n *for each of the three adder/subtrator units). These processing units are bounded by *O*(*n*). Therefore, the *n *× *n *dynamic programming r-mesh uses *O*(*n*^3^) processing units.

To handle all other scoring schemes, *k *× *n *adder/subtractor r-meshes and *n *× *n *max switches must be used. In addition, to avoid overflow (or underflow) all pre-defined pair-wise symbol matching scores may have to be scaled up (or down) so that the possible smallest (or largest) number can fit in the 1UN representation. With this configuration, the dynamic programming r-mesh takes *O*(*n*^4^) processing units.

#### Longest common subsequence (LCS)

The complication of signed numbers does not exist in the longest common subsequence problem. The arithmetic operation in LCS is a simple addition of 1 if there is a match. The same dynamic programming r-mesh as seen in Figure 6 can be used, where the two subtractors units are removed or the gap cost is set to zero (*g *= 0).

**Figure 7:**
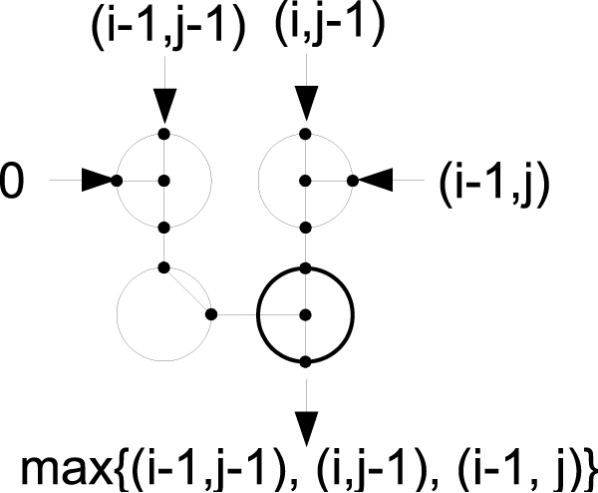
**A 4-way max switch**. A configuration of a 4-way max switch to solve the longest common subsequence (lcs). The South-East processing unit (in bold) configures {NS,E,W} if the symbols at row *i *and column *j *are different; otherwise, it configures{N,E,SW}. This figure show a configuration when the two symbols are different.

To find the longest common subsequence between two sequences *x *and *y*, each max switch in the DP r-mesh is configured as in Figure 7. The value from cell *c*_*i*-1, *j*-1 _is fed into the North-West processing unit, and the other values are fed into the North-East unit. Then, *c*_*i*, *j *_loads in its symbols and fuses the South-East processing unit (in bold) as NS,E,W if the symbols at row *i *and column *j *are different; otherwise, it loads 1 into the adder unit and fuses N,E,SW. These configurations allow either the value from cell *c*_*i*-1, *j*-1 _or the max value of cells *c*_*i*-1, *j *_and *c*_*i*, *j*-1 _to pass through. These are the only changes for the DP r-mesh to solve the LCS problems.

This modified constant-time DP r-mesh used *O*(*n*^3^) processing units. However, this is an order of reduction comparing the current best constant parallel DP algorithm that uses an r-mesh of size *O*(*n*^2^) × *O*(*n*^2^) [14] to solve the same problem.

### Affine gap cost

Affine gap cost (or penalty) is a technique where the opening gap has different cost from an extending gap [38]. This technique discourages multiple and disjoined gap insertion blocks unless their inclusion greatly improves the pair-wise alignment score. The gap cost is calculated as *p *= *o *+ *g*(*l *- 1), where *o *is the opening gap cost, *g *is the extending gap cost, and *l *is the length of the gap block. Traditionally, Gotoh use three matrices to track these values; however, it is not intercessory in the reconfigurable mesh computing model since each cell in the matrix is a processing node with local memory.

**Figure 8:**
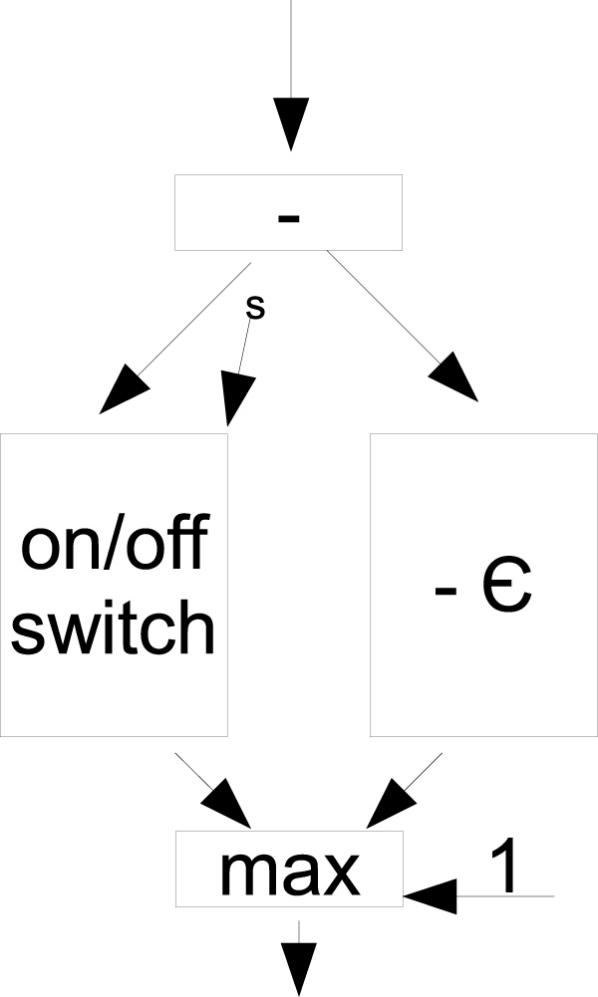
**A configuration for selecting a min value**. A configuration to select one of the two inputs in 1UN notation using the right most bit as a selector *s*. When *s *= 1 the switch is turned on to allow the data to flow through and get selected by the max switch. When the selector *s *= 0, the on/off switch produces zeros and the other data flow will be selected. ε = *o - g, o *≥ *g*, is the difference between opening gap cost *o *and extending gap cost *g*.

To handle affine gap cost, we need to extend the representation of the number by 1 bit (right most bit). This bit indicates whether a value coming from *c*_*i*-1, *j *_or *c*_*i*, *j*-1 _to *c*_*i*, *j *_is an opening gap or not. If the incoming value has been gap-penalized, its right most bit is 1, and it will not be charged with an opening gap again; otherwise, an opening gap will be applied. The original "-" units must be modified to accommodate affine gaps. Figure 8 shows the modification of the "-" unit. The output from the original "-" unit is piped into an *n *× *n *+ 1 r-mesh on/off switch (described in Section ), an adder/subtractor, and a max switch. When a number flows through the "-" unit, an extending gap is applied. If the incoming value has not been charged with gap to begin with, its right most bit (i.e. selector bit denoted as "s") remains zero, which keeps the switch in off position. Therefore, the value with extra charge on the adder/subtractor is allowed to flow through; otherwise, the switch will be on, and the larger value will be selected by the max switch. A value that is not from diagonal cells must have its selector bit set to 1 (right most bit) after a gap cost is applied to prevent multiple charges of an opening gap.

The modification of the dynamic programming r-mesh to handle affine gap cost requires additional 2 adder/subtractor units, 2 on/off switches, and one 2-input max switch. Asymptotically, the amount of processing units used is still bounded by *O*(*n*^4^) and the run-time complexity remains *O*(1).

### R-mesh on/off switches

**Figure 9:**
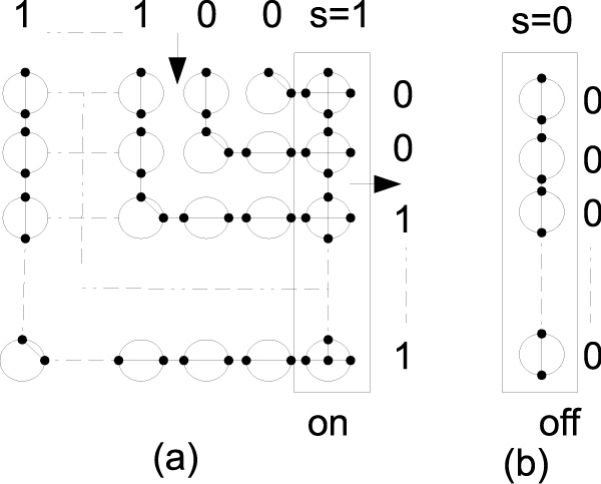
**An *n *× *n + *1 n-bit on/off switch**. By default, all processing units on the last column (column *n *+ 1) configure {NS,E,W}, and fuse {NSEW} when a signal (i.e. 1) travels through them. All cells on the main anti-diagonal cells of the first *n *rows and columns fuse {NE,S,W}, cells above the main anti-diagonal fuse {NS,E,W}, and cells below the main anti-diagonal fuse {N,S,EW}. Figure 9(a) shows the r-mesh configuration on a selector bit of 1 (s = 1) and Figure 9(b) shows the r-mesh configuration on a selector bit of 0 (s = 0).

To handle affine gap cost in dynamic programming, we need a switch that can select, i.e. turns on or off, the output ports of a data flow. The on/off r-mesh switch can be configured as in Figure 9, where the input value is mapped into the North-bound (top). The right most bit of the input is served as a selector bit. The r-mesh size is *n *× *n*+1, where column *i *fuses with row *n - i *to form an L-shape path that allows the input data from the Northbound to flow to the output port on the Eastbound. The processors on the last column will fuse the East-West ports allowing the input to flow through only if they receive a value of 1 from the input (Northern ports). Since the selector bit travels a shorter distance than all the other input bits, it will arrive in time to activate the opening or closing of the output ports.

This r-mesh configuration uses (*n *× *n *+ 1), i.e., *O*(*n*^2^), processing units to turn off the flow of an *n*-bit input in a broadcasting time.

### Dynamic programming back-tracking on r-mesh

The pair-wise alignment is obtained by following the path leading to the overall optimal alignment score, or the end of the alignment. In the case of the Needleman-Wunsch's algorithm, cell *c*_*n*, *n *_holds this value; and in the case of the Smith-Waterman's algorithm, cell *c*_*i*, *j *_with the maximum alignment score is the end point. The cell with the largest value can be located in *O*(1) time on a 3-dimension *n *× *n *× *n *r-mesh through these steps:

1. Initially, the DP matrix with calculated values are stored in the first slice of the r-mesh cube, i.e. in cells *c*_*i*, *j*,0_, 0 <*i*, *j *≤ *n*.

2. *c*_*i*, *j*,0 _sends its value to *c*_*i*, *j*, *i*_, 0 ≤ *i*, *j *≤ *n*, to propagate each column of the matrix to the 2D r-meshes on the third dimension.

3. *c*_*i*, *j*, *i *_sends its value to *c*_0, *j*, *k*_, i.e. to move the solution values to the first row of each 2D r-mesh slice.

4. Each 2D r-mesh slice finds its max value *c*_0, *r*, *k *_where *r *is the column of the max value in slice *k*

5. *c*_0, *r*, *k *_sends *r *to *c*_*k*,0,0_, i.e. each 2D r-mesh slice sends its max value column number *m *to the first 2D r-mesh slice. This value is the column index of the max value on row *k *in the first slice.

6. The first 2D r-mesh slice, *c*_*i*, *j*,0_, finds the max value of *n *DP r-mesh cells whose row index is *i *and column index is *c*_*i*0,0 _(i.e. value r received from the previous step). The row and column indices of the max value found in this step is the location of the max value in the original DP r-mesh.

These above steps rely on the capability to find the max value from *n *given numbers on an *n *× *n *r-mesh. This operation can be done in *O*(1) time as follows:

1. initially, the values are stored in the first row of the r-mesh.

2. *c*_0, *j *_broadcasts its value, namely *a*_*j*_, to *c*_*i*, *j*_, (column-wise broadcasting).

3. *c*_*i*, *i *_broadcasts its value, namely *a*_*i*_, to *c*_*i*, *j *_(row-wise broadcasting).

4. *c*_*i*, *j *_sets a flag bit *f*(*i*, *j*) to 1 if and only if *a*_*i *_>*a*_*j*_; otherwise sets *f*(*i*, *j*) to 0.

5. *c*_0, *j *_is holding the max value if *f*(0, *j*), *f*(1, *j*),..., *f*(*n *- 1, *j*) are 0. This step can be performed in *O*(1) time by ORing the flag bits in each column.

The location of the max value in the DP r-mesh can be obtained in *O*(1) time because each step in the process takes *O*(1) time to complete.

To trace back the path leading to the optimal alignment, we start with the end point cell *c*_*e*, *d *_found above and following these steps:

1. *c*_*i*, *j*_, (*i *≤ *e*, *i *≤ *d*), sends its value to *c*_*i*, *j*+1_, *c*_*i*+ 1, *j*_, *c*_*i*+1, *j+i*_. Thus, each cell can receive up to three values coming from its Noth, West, and Northwest borders.

2. *c*_*i*, *j *_finds the max of the inputs and fuses its port to the neighbor cell that sent the max value in the previous step. If there are more than one port to be fused, (this happens when there are multiple optimal alignments), *c*_*i*, *j *_randomly selects one.

3. *c*_*e*, *d *_sends a signal to its fused port. The optimal pair-wise alignment is the ordered list of cells where this signal travels through.

Each operation in the back-tracking process takes *O*(1) time to complete, and there are no iterative operations. Therefore, the back-tracking steps requires *n*^3 ^processing units and takes *O*(1) time.

## Progressive multiple sequence alignment on r-mesh

In this section, we start by describing a parallel algorithm to generate a dendrogram, or guiding tree, representing the order in which the input sequences should be aligned. Then we will show a reworked version of sum-of-pair scoring method that can be performed in constant time on a 2D r-mesh. Finally, we will describe our parallel progressive multiple sequence alignment algorithm on r-mesh along with its complexity analysis.

### Hierarchical clustering on r-mesh

The parallel neighbor-joining (NJ) [12] clustering method on r-mesh is described here; other hierarchical clustering mechanisms can be done in a similar fashion. The neighbor-joining takes a distance matrix between all the pairs of sequences and represents it as a star-like connected tree, where each sequence is an external node (leaf) on the tree. NJ then finds the shortest distance pair of nodes and replaces it with a new node. This process is repeated until all the nodes are merged.

Followings are the actual steps to build the dendrogram:

1. Initially, all the pair-wise distances are given in form of a matrix *D *of size *m *× *m*, where m is the number of nodes (or input sequences).

2. Calculate the average distance from node *i *to all the other nodes by $r_{i}=\frac{\sum_{1}^{m}D_{ij}}{m-2}$.

3. The pair of nodes with the shortest distance (*i*, *j*) is a pair that gives minimal value of *M*_*ij*_, where *M*_*ij *_= *D*_*ij *_- *r*_*i *_- *r*_*j*_.

4. A new node *u *is created for shortest pair (*i,j*), and the distances from *u *to *i *and *j *are: $d_{iu}=\frac{D_{ij}}{2}+\frac{\left(r_{i}-r_{j}\right)}{2}$, and *d*_*j,u *_= *d*_*ij*_-*d*_*iu*_.

5. The distance matrix *D *is updated with the new node *u *to replace the shortest distance pair (*i,j*), and the distances from all the other nodes to *u *is calculated as *D*_*vu *_= *D*_*iv *_+ *d*_*jv *_- *D*_*ij*_.

These steps are repeated for *m *- 1 iterations to reduce distance matrix *D *to one pair of nodes. The last pair does not have to be merged, unless the actual location of the root node is needed.

Step 1 and 4 are constant time operations on an *m *× *m *r-mesh, where each processing unit stores a corresponding value from the distance matrix. Since the progressive multiple sequence alignment algorithm only uses the dendrogram as a guiding tree to select the closest pair of sequences (or two groups of sequences), the actual distance values between the nodes on the final dendrogram mostly are insignificant. Consequently, the values in distance matrix *D *can be scaled down without changing the order of the nodes in the dendrogram. In addition, if these values are not to be preserved, the calculations in step 4 can be skipped.

Before proceeding to step 2, we should reexamine some facts. First, the maximum alignment score from all the pair-wise DP sequence alignments are bounded by *b*^2^, where *b *is the max pair-wise score between any two residue symbols. An alignment score of *b*^2 ^occurs only if we align a sequence of these symbols to itself. *b*+1 ≤ *n *is the number of bits being used to represent this value in 1UN. Similarly, the maximum value in distance matrix *D *generated from these alignment scores are also bounded by *b*^2^. Thus, the sum of *n *of these distance values are bounded by *b*^4^. These facts allow us to calculate the sums in step 2 in *O*(*c*) time using an *n *× *n *r-mesh as in Theorem 1. In this case, c is constant, (*c *= 4). There are *n *summations to calculate, so the entire calculation requires *n *such r-meshes, or *n*^3 ^processing units, to complete in *O*(1) time.

In step 3, each processing unit computes value *M*_*ij *_locally. The max value can be found using the same technique described in Section in constant time.

Similarly, step 5 is performed locally by the processing units in the r-mesh in *O*(1) time. Since this procedure terminates after *m *- 1 iterations, the overall run-time complexity to build a dendrogram, (or guiding tree), for any given pair-wise distance matrix of size *m *× *m *is *O*(*m*) using *O*(*m*3) processing units.

### Constant run-time sum-of-pair scoring method

The third step [step (iii)] of the progressive MSA algorithm is following the dendrogram, built in the earlier step, to perform pair-wise dynamic programming alignment on two pre-aligned groups of sequences. The dynamic programming alignment algorithm in this step is exactly the same as the one in step (i); however, quantifying a match between two columns of residues are no longer a simple constant look-up, unless the hierarchical expected probability (HEP) matching scoring scheme is used [39]. The most popular quantifying method is the sum-of-pair (SP) method [40], or its variations as seen in [5-7,41]. This quantification is the sum of all pair-wise matching scores between the residue symbols, where each paired-score is obtained either from a substitution matrix or from any scoring scheme discussed earlier. The alignment at the root of the tree gets *n *residues for every pair of columns to be quantified. Thus, there are $\frac{m\left(m-1\right)}{2}$ lookups per column quantification, i.e. $\frac{m\left(m-1\right)}{2}$ lookups or each DP matrix cell calculation. The sum-of-pair is formally defined as:

(1) $$sp\left(f,g\right)=\overset{\left|f\right|}{\underset{i=1}{\sum }}\overset{\left|g\right|}{\underset{j=i+1}{\sum }}s\left(f_{i},g_{j}\right)$$

where *f *is a column from one pre-aligned group of sequences and *g *is a column from the other pre-aligned group of sequences. *f*_*i *_and *g*_*j *_are residue symbols from columns *f *and *g*, respectively, and *s*(*f*_*i*_, *g*_*j*_) is the matching score between these two symbols *f*_*i *_and *g*_*j*_. For example, to calculate the sum-of-pair of the following two columns *f *and *g*:

$$\text{Column }f:\left\{\begin{array}{c} A\\ C\\ T \end{array}\right\}$$

and

$$\text{Column }g:\left\{\begin{array}{c} G\\ T\\ T \end{array}\right\}$$

we will have to score 15 residue pairs:(A,C), (A,T), (A,G), (A,T), (A,T), (C,T), (C,G), (C,T), (C,T), (T,G), (T,T), (T,T), (G,T), (G,T), (T,T). Since the matching between residue *a *to residue *b *is the same as the matching between residue *b *to residue *a*, these pairs become (A,C), 3(A,T), (A,G), 3(C,T), (C,G), 3(G,T), 3(T,T). These redundancies occur since the set of symbols representing the residues is small (1 for gap plus 20 for protein [or 4 for DNA/RNA]). Thus, if we combine the two column symbols with their number of occurrences, the sum-of-pair method can be transformed into a counting problem and can be defined as:

(2) $$sp\left(f,g\right)=\overset{T}{\underset{i=1}{\sum }}\frac{n_{i}\left(n_{i}-1\right)}{2}s\left(i,i\right)+n_{i}\overset{T}{\underset{j=i+1}{\sum }}n_{j}\times s\left(i,j\right)$$

where *f*, *g *are the two columns, *T *is the number of different residue symbols (T = 4 for DNA/RNA and T = 20 for proteins), *s*(*i,j*) is the pair-wise matching score, or substitution score, between two residue symbols *i *and *j*, and *n*_*i *_and *n*_*j *_are the total count of symbols/types *i *and *j *(i.e. the occurrences of residue symbols/types *i *and *j*), respectively. Since residues from both column *f *and *g *are merged, there is no distinction in which column the residue are from. Since *T *is constant, the summations in Equation remain constant, regardless how many sequences are involved.

Thus, the sum-of-pair score of the two columns given above will be:

$$\frac{3\left(3-1\right)}{2}\text{s}\left(\text{T},\text{T}\right)+\left[\text{s}\left(\text{A,C}\right)+\text{s}\left(\text{A,G}\right)+3\text{s}\left(\text{A,T}\right)+\text{s}\left(\text{C,G}\right)+3\text{s}\left(\text{C,T}\right)+3\text{s}\left(\text{G,T}\right)\right]$$

This scoring function can be implemented on an array of *m *processing units as follows. First, map each residue symbol into a numeric value from 1 to *T*. Next, *m *residues from any two aligning columns are assigned to *m *processing units. Any processing unit holding a residue sends a 1 to processing unit *p*_*k*_, where *k *is the number represents the residue symbol it is holding. *p*_*k *_sums the 1's it receives. The sum-of-pair score can be computed between the pairs of processing units containing a sum larger than 0 calculated from previous steps. All of these steps are carried out in constant time. There are *n*^2 ^possible pair-wise column arrangements of two pre-aligned groups of sequences of max length *n*. Thus, the sum-of-pair column pair-wise matching scores for two pre-aligned groups of sequences can be done in *O*(1) using *m *× *n*^2 ^processing units.

### Parallel progressive MSA algorithm and its complexity analysis

Progressive multiple sequence alignment algorithm is a heuristic alignment technique that builds up a final multiple sequence alignment by combining pair-wise alignments starting with the most similar pair and progressing to the most distant pair. The distance between the sequences can be calculated by dynamic programming algorithms such as Smith-Waterman's or Needle-Wunsch's algorithms (step i). The order in which the sequences should be aligned are represented as a guiding and can be calculated via hierarchical clustering algorithms similar to the one described in Section (step ii). After the guiding tree is completed, the input sequences can be pair-wise aligned following the order specified in the tree (step iii). In the previous Sections, we have described and designed several r-meshes to handle individual operations in the progressive multiple alignment algorithm. Finally, a progressive multiple sequence alignment r-mesh configuration can be constructed. First, the input sequences are pair-wise aligned using the dynamic programming r-mesh described previously in Section. These $\frac{m\left(m-1\right)}{2}$ pair-wise alignments can be done in *O*(1) using $\frac{m\left(m-1\right)}{2}$ dynamic programming r-meshes, or in *O*(*m*) time using *O*(*m*) r-meshes. The latter is preferred since the dendrogram [step (ii)] and the progressive alignment [step (iii)] each takes *O*(*m*) time to complete. Then, a dendrogram is built, using the parallel neighbor-joining clustering algorithm described earlier, from all the pair-wise DP alignment scores from step (i). Lastly, [step (iii)], for each pair of pre-aligned groups of sequences along the dendrogram, the sum-of-pair column matching scores are pre-calculated for the DP r-mesh initialization before proceeding with the dynamic programming alignment. There are *m *- 1 branches in the dendrogram leading to *m *- 1 pair-wise group alignments to be performed. In terms of complexity, the progressive multiple sequence alignment takes *O*(*m*) time using *O*(*n*) DP r-meshes to complete all the pair-wise sequence alignments [step (i)] - (or *O*(1) time using $\frac{m\left(m-1\right)}{2}$ DP r-meshes). Its consequence step, [step (ii)], to build the sequence dendrogram takes *O*(*m*) time using *O*(*m*^3^) processing units. Finally, the progressive step, [step (iii)], takes O(m) time using a DP r-mesh. Therefore, the overall run-time complexity of this parallel progressive multiple sequence alignment is *O*(*m*). The number of processing units utilized in this parallel algorithm is bounded by the number of DP r-meshes used and their sizes. The general DP r-mesh uses *O*(*n*^4^) processing units to handle all scoring schemes with affine gap cost. And step (i) needs *m *of such DP r-meshes resulting in *O*(*mn*^4^) ≈ *O*(*n*^5^) processing units used.

For alignment problems that use constant scoring schemes without affine gap cost, this parallel progressive multiple sequence alignment algorithm only needs *O*(*mn*^3^) ≈ *O*(*n*^4^) processing units to complete in *O*(*m*) time.

Table 1 summarizes the r-mesh size and the run-time complexity of various components in this study, where the components with "broadcast" run-time can finish their operations in one broadcasting time. The "DP" r-mesh is designed to handle all the Needleman-wunsch's [10], Smith-Waterman's [9], and Longest Common Subsequence algorithms.

**Table 1:** Summary of progressive multiple sequence alignment components

Component	input size	processors	run-time
2-input max switch	1 - *bit*	1	1 broadcast
4-input max switch	1 - *bit*	4	1 broadcast
2-input max switch	*n *- *bit*	*n*	1 broadcast
4-input max switch	*n *- *bit*	4*n*	1 broadcast
on/off switch	*n *- *bit*	*n *×*n *+1	1 broadcast
adder/subtractor	*n*	*k *×*n*, *k *≤ *n*	1 broadcast
DP(const. scoring)	2 sequences, max length = *n*	*O*(*n*^3^)	1 broadcast
DP (general scoring)	2 sequences, max length = *n*	*O*(*kn*^3^), *k *≤ *n*	1 broadcast
DP back-tracking	*n *× *n*	*n *× *n *× *n*	*O*(1)
Neighbor-Joining	*m *× *m*	*O*(*m*^3^)	*O*(*m*)
Sum-of-pair	2 pre-aligned groups of m sequences	*m *× *n*^2^	*O*(1)
MSA(const. scoring)	*m *sequences, max length = *n*	*O*(*m *× n^3^)	*O*(*m*)
MSA	*m *sequences, max length = *n*	*O*(*m *× n^4^)	*O*(*m*)

This Table summarizes all the parallel components developed in this study along with their time and CPU complexity.

## Conclusions

In this study, we have designed various r-mesh components that can run in one broadcasting step, which enabling us to effectively parallelize the progressive multiple sequence alignment paradigm. to align *m *sequences with max length *n*, we are able to reduce the algorithm run-time complexity from *O*(*m *× *n*^4^) to *O*(*m*) using *O*(*m *× *n*^4^) processing units. For a scoring scheme that rewards 1 for a match, 0 for a mismatch, and -1 for a gap insertion/deletion, our algorithm uses only *O*(*m *× *n*^3^) processing units. Moreover, to our knowledge, we are the first to propose an *O*(1) run-time dynamic programming pair-wise alignment algorithm using only *O*(*n*^3^) processing units.

## Competing interests

The authors declare that they have no competing interests.

## Authors' contributions

KN designed parallel models used in this study. YP and GN participated in designing and criticizing the parallel models and their analysis. All authors read and approved the final manuscript.

## References

[B1] Rosenberg MS (Ed) Sequence alignment - methods, models, concepts, and strategies 2009 University of California Press

[B2] Wang L Jiang T On the complexity of multiple sequence alignment J Comput Biol 1994 1 4 337 48 10.1089/cmb.1994.1.337 8790475

[B3] Dayhoff MO Schwartz RM Orcutt BC A model of evolutionary change in proteins: matrices for detecting distant relationships Atlas of Protein Sequence and Structure 1978 5 Suppl 3 345 358

[B4] Henikoff S Henikoff JG Amino acid substitution matrices from protein blocks Proceedings of the National Academy of Sciences 1992 89 22 10915 10919 10.1073/pnas.89.22.10915 PMC50453 1438297

[B5] Thompson J Higgins D Gibson T CLUSTAL W: improving the sensitivity of progressive multiple sequence alignment through sequence weighting, position-specific gap penalties and weight matrix choice Nucleic Acids Res 1994 22 22 4673 4680 10.1093/nar/22.22.4673 7984417 PMC308517

[B6] Do C Mahabhashyam M Brudno M Batzoglou S ProbCons: probabilistic consistency-based multiple sequence alignment Genome Res 2005 15 330 340 10.1101/gr.2821705 15687296 PMC546535

[B7] Notredame C Higgins D Heringa J T-Coffee: a novel method for fast and accurate multiple sequence alignment J Mol Biol 2000 302 205 217 10.1006/jmbi.2000.4042 10964570

[B8] Nguyen KD Pan Y Multiple sequence alignment based on dynamic weighted guidance tree International Journal of Bioinformatics Research and Applications 2011 7 2 168 182 10.1504/IJBRA.2011.040095 21576075

[B9] Smith TF Waterman MS Identification of common molecular subsequences Journal of Molecular Biology 1981 147 195 197 10.1016/0022-2836(81)90087-5 7265238

[B10] Needleman SB Wunsch CD A general method applicable to the search for similarities in the amino acid sequence of two proteins J Mol Biol 1970 48 3 443 53 10.1016/0022-2836(70)90057-4 5420325

[B11] Sneath PHA Sokal RR Numerical taxonomy. The principles and practice of numerical classification 1973 Freeman, San Francisco 573

[B12] Saitou N Nei M The neighbor-joining method: a new method for reconstructing phylogenetic trees 1987 4 Oxford University Press 406 425 10.1093/oxfordjournals.molbev.a040454 3447015

[B13] Lipman D Altschul S Kececioglu J A Tool for multiple sequence alignment Proceedings of the National Academy of Sciences 1989 86 12 4412 4415 10.1073/pnas.86.12.4412 PMC287279 2734293

[B14] Bertossi AA Mei A Constant time dynamic programming on directed reconfigurable networks IEEE Transactions on Parallel and Distributed Systems 2000 11 529 536 10.1109/71.862204

[B15] Huang CH Biswas R Parallel pattern identification in biological sequences on clusters Cluster Computing, IEEE International Conference on 2002 0 127

[B16] Lee HC Ercal F R-mesh algorithms for parallel string matching Third International Symposium on Parallel Architectures, Algorithms, and Networks, I-SPAN '97 Proceedings 1997 223 226

[B17] Lima CRE Lopes HS Moroz MR Menezes RM Multiple sequence alignment using reconfigurable computing ARC'07: Proceedings of the 3rd international conference on Reconfigurable computing 2007 Berlin, Heidelberg: Springer-Verlag 379 38422121496

[B18] Liu Y Schmidt B Maskell DL MSA-CUDA: multiple sequence alignment on graphics processing units with CUDA Application-Specific Systems, Architectures and Processors, IEEE International Conference on 2009 0 121 128

[B19] Sarkar S Kulkarni GR Pande PP Kalyanaraman A Network-on-Chip hardware accelerators for biological sequence alignment IEEE Transactions on Computers 2010 59 29 41

[B20] Raju VS Vinayababu A Optimal parallel algorithm for string matching on mesh network structure International Journal of Applied Mathematical Sciences 2006 3 167 175

[B21] Raju VS Vinayababu A Parallel algorithms for string matching problem on single and two-dimensional reconfigurable pipelined bus systems Journal of Computer Science 2007 3 754 759

[B22] Takefuji Y Tanaka T Lee K A parallel string search algorithm Systems, Man and Cybernetics, IEEE Transactions on 1992 22 2 332 336 10.1109/21.148407

[B23] Oliver T Schmidt B Nathan D Clemens R Maskell D Using reconfigurable hardware to accelerate multiple sequence alignment with ClustalW Bioinformatics 2005 21 16 3431 3432 http://bioinformatics.oxfordjournals.org/content/21/16/3431.abstract 10.1093/bioinformatics/bti508 15919726

[B24] Oliver T Schmidt B Maskell D Nathan D Clemens R High-speed multiple sequence alignment on a reconfigurable platform Int J Bioinformatics Res Appl 2006 2 394 406 http://portal.acm.org/citation.cfm?id=1356527.1356532 10.1504/IJBRA.2006.011038 18048180

[B25] Huang X A space-efficient parallel sequence comparison algorithm for a message-passing multiprocessor Int J Parallel Program 1990 18 3 223 239

[B26] Aluru S Futamura N Mehrotra K Parallel biological sequence comparison using prefix computations Journal of Parallel and Distributed Computing 2003 63 3 264 272 http://www.sciencedirect.com/science/article/B6WKJ-48CFNBJ-3/2/e9cbdc3abeab30a9b1912cd5d7802331 10.1016/S0743-7315(03)00010-8

[B27] Dally W Towles B Route packets, not wires: on-chip interconnection networks Journal of Parallel and Distributed Computing 2001 684 689

[B28] Tan G Feng S Sun N Parallel multiple sequences alignment in SMP cluster HPCASIA '05: Proceedings of the Eighth International Conference on High-Performance Computing in Asia-Pacific Region 2005 IEEE Computer Society 426

[B29] Luo J Ahmad I Ahmed M Paul R Parallel multiple sequence alignment with dynamic scheduling ITCC '05: Proceedings of the International Conference on Information Technology: Coding and Computing (ITCC'05) 2005 I Washington, DC, USA: IEEE Computer Society 8 1322119199

[B30] Miller R Prasanna VK Reisis DI Stout QF IEEE Trans. Computers Parallel computations on reconfigurable meshes

[B31] Shi H Ritter GX Wilson JN Simulations between two reconfigurable mesh models Information Processing Letters 1995 55 3 137 142 http://www.sciencedirect.com/science/article/B6V0F-3YYTDS7-15/2/1443b1ec225f2536c578ed52f1143cfa 10.1016/0020-0190(95)00082-N

[B32] Pan Y Li K Hamdi M An improved constant-time algorithm for computing the Radon and Hough transforms on a reconfigurable mesh Systems, Man and Cybernetics, Part A: Systems and Humans, IEEE Transactions on 1999 29 4 417 421 10.1109/3468.769762

[B33] Bourgeois AG Trahan JL Relating two-dimensional reconfigurable meshes with optically pipelined buses Parallel and Distributed Processing Symposium, International 2000 0 747

[B34] Trahan JL Bourgeois AG Pan Y Vaidyanathan R Optimally scaling permutation routing on reconfigurable linear arrays with optical buses Journal of Parallel and Distributed Computing 2000 60 9 1125 1136 http://www.sciencedirect.com/science/article/B6WKJ-45F4YHC-X/2/7749ae137af49ed1a8b374762b7d0d67 10.1006/jpdc.2000.1643

[B35] Nguyen KD Bourgeois AG Ant colony optimal algorithm: fast ants on the optical pipelined r-mesh International Conference on Parallel Processing (ICPP'06) 2006 347 354

[B36] Cordova-Flores CA Fernandez-Zepeda JA Bourgeois AG Constant time simulation of an r-mesh on an lr-mesh Parallel and Distributed Processing Symposium, International 2007 0 269

[B37] Vaidyanathan R Trahan JL Dynamic reconfiguration: architectures and algorithms 2004 Kluwer Academic/Plenum Publishers

[B38] Gotoh O An improved algorithm for matching biological sequences Journal of Molecular Biology 1982 162 3 705 708 http://www.sciencedirect.com/science/article/pii/0022283682903989 10.1016/0022-2836(82)90398-9 7166760

[B39] Nguyen KD Pan Y A reliable metric for quantifying multiple sequence alignment Proceedings of the 7th IEEE international conference on Bioinformatics and Bioengineering (BIBE 2007) 2007 788 795

[B40] Carillo H Lipman D The multiple sequence alignment problem in biology SIAM Journal of Applied Math 1988 48 5 1073 1082 10.1137/0148063

[B41] Katoh K Misawa K Kuma K Miyata T MAFFT: a novel method for rapid multiple sequence alignment based on fast Fourier transform Nucleic Acids Research 2002 30 14 3059 3066 10.1093/nar/gkf436 12136088 PMC135756

